# Metacognitive Ratings on Cognitive Tasks: Task Difficulty and Effort Rating Differences in Children With ADHD and Neurotypical Children

**DOI:** 10.1177/10870547261428879

**Published:** 2026-03-26

**Authors:** Adrian P. Torres, Maggie E. Toplak

**Affiliations:** 1 York University, Toronto, ON, Canada; 2 LaMarsh Centre for Child and Youth Research, Toronto, ON, Canada

**Keywords:** metacognition, effort, ADHD, performance-based tasks, children

## Abstract

**Objective:**

The role of metacognitive monitoring, or the subjective evaluation of performance during cognitive tasks, has been less well studied in children with ADHD compared to accuracy or performance on these tasks. Given that children with ADHD often display lower performance on cognitive tasks, particularly those involving executive attention and control, we examined whether metacognitive monitoring differed between children with ADHD and neurotypical children.

**Method:**

Eighty children aged 8–12 years (38 with ADHD, 42 neurotypical) completed a battery of cognitive tasks, including measures of intelligence, executive functioning (set-shifting and interference control), and an unstructured performance task (UPT). After each task, participants provided ratings of task difficulty and effort (how hard they tried).

**Results and Conclusion:**

Children with ADHD reported overall lower effort across cognitive tasks compared to neurotypical children; however, no group differences were found on task difficulty ratings. Metacognitive ratings of effort were significantly associated across performance tasks, suggesting that the degree of trying may reflect a trait-level factor. Ratings of task difficulty were generally weakly associated, suggesting ratings are influenced by task-specific factors. Metacognitive ratings of effort and task difficulty were not correlated, and these ratings were also distinct from task performance, with generally weak, non-significant associations. Metacognitive ratings of effort, not ratings of task difficulty, predict difficulties experienced by children with ADHD, beyond what is captured by performance on these tasks alone.

## Introduction

There is an extensive literature on differences between individuals with Attention-Deficit/Hyperactivity Disorder (ADHD) and neurotypical groups on several measures related to cognitive performance, including attention, intellectual abilities, and executive control (EC) or executive functions (EF; Dennis et al., 2009; Frazier et al., 2004; Schachar, 2023; Sjöwall et al., 2013; Willcutt et al., 2005) and on their clinical utility in the assessment of ADHD (Arrondo et al., 2024; Bellato et al., 2024; Sawaya et al., 2024). However, metacognitive monitoring has been less studied than cognitive performance, particularly executive functioning, in individuals with ADHD (Wanstall et al., 2019). ADHD is a neurodevelopmental disorder characterized by difficulties with hyperactivity, inattention, and impulsive behavior (DSM-5-TR; American Psychiatric Association [APA], 2022). Given that ADHD is characterized by difficulties in sustaining attention and regulating behavior, examining paradigms that provide methods to assess metacognitive monitoring in individuals with ADHD could further operationalize these difficulties. Thus, the purpose of the current study was to examine metacognitive ratings of task difficulty and effort after completing cognitive tasks in a sample of children with ADHD and a neurotypical comparison group. Specifically, we examined whether these ratings differed between ADHD and neurotypical groups, whether they varied systematically across the four different cognitive tasks (intelligence, interference control, set shifting, and an unstructured performance task), and whether they were associated with task performance.

Metacognition is defined as the ability to monitor and regulate one’s cognitive processes (Dunlosky & Metcalfe, 2009; Pintrich, 2002) and is crucial for determining the level of engagement needed for successful task completion (Fischer et al., 2014; Scherbaum et al., 2011; Shenhav et al., 2021). Metacognitive paradigms typically involve participants’ performance on cognitive tasks and subjective ratings of confidence in their performance and judgments of learning, which are then compared with their actual success in the task (Bjork et al., 2013). The ability to accurately assess one’s performance and knowledge while completing a task is crucial in order to make decisions, such as whether to provide an answer, give up, or allocate further investment of effort (Bjork et al., 2013; Fiedler et al., 2019). Effective monitoring of performance has been suggested to explain why adjusting actions and behavior may be difficult for individuals with ADHD (DSM-5-TR, APA, 2022; Gallagher & Blader, 2001).

Although there has been interest in how individuals with ADHD perceive their difficulties, most empirical methods have used comparisons between self- and informant-reports (e.g., Jiang & Johnston, 2017; Watabe et al., 2018). These studies often reveal a Positive Illusory Bias (PIB) among children and adolescents with ADHD, who tend to rate themselves more favorably regarding executive functioning, ADHD symptoms, academics, and social and behavioral areas compared to ratings from teachers and parents, as well as relative to neurotypical controls (Hoza et al., 2004; Steward et al., 2017; Volz-Sidiropoulou et al., 2016; Whitley et al., 2008; Wiener et al., 2012). This phenomenon has been studied in different domains, from driving ability (Knouse et al., 2005; Weafer et al., 2008) to performance on attention tasks (Butzbach et al., 2021). Across these studies, it has been suggested that individuals with ADHD may be less accurate in how they perceive their functioning relative to other informants reporting on their functioning.

Relatively few studies in the field of ADHD have examined differences in metacognitive ratings when referenced to an objective performance-based marker (Butzbach et al., 2021; Knouse et al., 2005, 2006, 2012; Mies et al., 2019; Prevatt et al., 2011; Wanstall et al., 2019; Weafer et al., 2008). Some studies conducted on children and adolescents with ADHD have reported differences in metacognitive monitoring (Fliers et al., 2010; Helseth et al., 2016; Hoza et al., 2000; Owens & Hoza, 2003), particularly overestimation of performance across various domains (Hoza et al., 2000, 2001; Milich & Okazaki, 1991; Ohan & Johnston, 2002; Owens & Hoza, 2003). For example, Hoza et al. (2001) found that boys with ADHD reported self-evaluations of performance on a find-a-word task similar to controls despite performing worse. Similar findings have been observed when using objective markers such as physical abilities (Helseth et al., 2016), achievement tests (Owens & Hoza, 2003), metamemory tasks (Antshel & Nastasi, 2008; Castel et al., 2011), and social interaction tasks (Hoza et al., 2000). Similar differences have also been reported in girls with ADHD compared to neurotypical girls (Ohan & Johnston, 2011). However, some studies have found no significant differences in judgments of learning and predictions of performance on metamemory tasks (Knouse et al., 2006, 2012).

While studies on ADHD and metacognition have primarily focused on perceptions of competence related to performance or behavior, the purpose of the current study was to examine subjective ratings of task difficulty and effort (i.e., how hard participants tried) across multiple cognitive tasks. The current study extends research on metacognition in children with ADHD by incorporating methods from the metacognitive literature, including obtaining subjective ratings immediately after each cognitive task to provide a specific reference point for evaluation.

### Metacognitive Ratings: Task Difficulty and Effort

Generally, metacognitive accuracy has been measured by assessing the difference between ratings of confidence or perceived performance and objective measures of cognitive performance (Akturk & Sahin, 2011; Fleming & Frith, 2014; Georghiades, 2004; Koriat & Goldsmith, 1996; Maniscalco & Lau, 2012; Nelson, 1990; Yeung & Summerfield, 2012). However, metacognitive monitoring also involves the perceived demands of the task itself (task difficulty) and how hard one tried on the task, reflecting the deliberate effort the individual invested to succeed (Hsu et al., 2017; F. G. W. C. Paas, 1992). According to Cognitive Load Theory (CLT), cognitive load represents the demands that performing a particular task imposes on the learner’s cognitive system, with an emphasis on working memory limitations (F. Paas & Van Merriënboer, 1994; Sweller et al., 2019). Self-report appraisals of effort and task difficulty are often used in cognitive load research as indicators of resource allocation to meet task demands (Brünken et al., 2003, 2006; F. G. W. C. Paas, 1992; Naismith et al., 2015; van Gog & Paas, 2008). These ratings aim to capture how individuals perceive and respond to cognitive demands, helping to quantify both task-driven and voluntary effort within a task context.

However, ratings of task difficulty and effort (i.e., how hard one tried) have been shown to be distinct. For instance, across three different cognitive tasks, the correlation between task difficulty ratings and accuracy was higher than the correlation between effort ratings and accuracy (Hoch et al., 2023). Similarly, Mies et al. (2019) reported that ratings of task difficulty increased with objective task difficulty, whereas ratings of how much participants did their best showed the opposite pattern in a sample of boys with ADHD and neurotypical children. Recent work by Schuessler et al. (2024) further shows that, although ratings of task difficulty and invested mental effort are strongly related, they are not identical constructs. Together, these findings support the view that invested effort and perceived task difficulty tap distinct aspects of subjective cognitive load. Consistent with this, van Gog and Paas (2008) argued that difficulty ratings primarily reflect the perceived demands of the task, whereas effort ratings index the amount of cognitive resources learners actually invest to meet those demands. Drawing on metacognitive accounts integrated with CLT, ratings of effort and task difficulty can be conceptualized in terms of goal-driven and data-driven effort (Koriat et al., 2006; Scheiter et al., 2020). Goal-driven effort, reflecting voluntary investment based on individual motivation, aligns with top-down processing, whereas data-driven effort reflects the perceived cognitive demands imposed by the task itself and is closely related to ratings of task difficulty. Thus, ratings of task difficulty and effort appear to be empirically separable and may involve distinct processing mechanisms.

Several studies have been conducted to explore how individuals with ADHD perceive cognitive demands. For instance, Hsu et al. (2017) found that individuals at risk for ADHD reported higher ratings of real-time mental effort and discomfort than a non-risk group on working memory tasks. Brown et al. (2020) found that higher reported ratings of volitionally exerted effort on a multimedia instruction task were associated with increased ADHD symptoms. However, in a pilot study evaluating behavioral, physiological, and subjective responses on the N-back task (working memory task), no differences in the rating of how mentally demanding the task was (cognitive demand) or how much participants reported trying their best (applied effort) were observed between adolescent boys with ADHD and controls (Mies et al., 2019). These studies suggest that individuals with ADHD may report greater mental effort and discomfort during cognitive tasks relative to neurotypical controls; evidence for group differences in perceived task difficulty is limited, and findings for volitionally exerted effort are mixed. In the current study, we examined ratings of both task difficulty and volitionally exerted effort on four cognitive tasks in a sample of ADHD and neurotypical groups.

A set of four cognitive tasks was chosen to sample a range of tasks that varied in demands and difficulty. Specifically, we included measures of intelligence (verbal and nonverbal), two executive function tasks (Stroop to assess interference control and Trail Making to assess set shifting), and an experimental task to assess performance under less-structured conditions (Unstructured Performance Task; Ledochowski et al., 2019). All of these tasks have been examined in samples of children with ADHD, who generally show poorer performance on these measures than typically developing peers (Bain & Jaspers, 2010; Frazier et al., 2004; Ledochowski et al., 2019; van Mourik et al., 2005; Willcutt et al., 2005). Based on findings from Mulert et al. (2007), ratings of “how hard one tried” remain relatively constant regardless of task difficulty, suggesting that effort investment may be more consistently related across different levels of difficulty and may reflect trait-level factors (Kramer et al., 2021; Kührt et al., 2021). Ratings of task difficulty across tasks were expected to show only modest associations, with the strongest correlations between tasks that share similar EF demands (Trail Making and Stroop). In contrast, ratings of “how hard I tried” were expected to correlate strongly across tasks, reflecting a more trait-like, person-level tendency to invest effort.

Thus, the present study expands on the limited research examining metacognitive ratings of task difficulty and effort (i.e., how hard one tried) in ADHD samples. We included a broad set of cognitive measures, including intelligence, executive function tasks (set shifting and interference control), and an unstructured performance task (UPT) in a sample of children with ADHD and neurotypical children. We compared metacognitive ratings of task difficulty and effort across the performance tasks and between groups. We expected overall group differences on both task difficulty and effort ratings, with those in the ADHD group reporting lower effort ratings and higher task difficulty ratings compared to the neurotypical group. Similar to previous findings, we predicted that effort ratings would be strongly associated across performance tasks, whereas difficulty ratings would not. Lastly, we predicted weak associations between task difficulty and effort ratings across tasks, reflecting distinct metacognitive judgments.

## Methods

### Participants

The sample comprised eighty children aged 8 to 12 years (*M* = 9.56 years, *SD* = 1.29, 26 females) and their caregivers. Children in the ADHD group (*n* = 38) were recruited from an outpatient mental health service, private psychological practices, and advertising on a website that provides education for families who have children with ADHD (*M* = 9.55 years, *SD* = 1.37; 11 girls and 27 boys). Children in the neurotypical group (*n* = 42) were recruited by advertising the study in the community (*M* = 9.57 years, *SD* = 1.23; 15 girls and 27 boys).

The inclusion criteria for the ADHD group were as follows: (1) prior diagnosis of ADHD; (2) meeting diagnostic criteria for ADHD on the Computerized Diagnostic Interview Schedule for Children–Parent Version (C-DISC; Fisher et al., 2006); and (3) *t*-score ≥70 on the Child Behavior Checklist Attention Scale completed by parents (CBCL; Achenbach & Rescorla, 2001). The inclusion criteria for the neurotypical group were: (1) absence of prior diagnosis of ADHD; and (2) not meeting the diagnostic criteria for ADHD on the C-DISC.

Half of the participants in the ADHD group were taking psychotropic medication (*n* = 19; 50%). In the ADHD group, eighteen participants had a diagnosis of Oppositional Defiant Disorder (47.4%), ten participants had a diagnosis of a Learning Disorder (26.3%), two participants had a diagnosis of a Language Impairment (5.3%), one participant had a diagnosis of a Conduct Disorder (2.6%), and three participants had both Oppositional Defiant and Conduct Disorder (7.9%). All parents of the neurotypical children reported no use of psychotropic medication. One participant in the neurotypical group was identified with a Learning disorder, and another was identified with a language impairment. Participants from both groups had an intelligence quotient (IQ) at or above the borderline range (IQ ≥ 70) on the Kauffman Brief Intelligence Test, Second Edition (KBIT-2; see section below for a description of this measure). The ADHD group had an estimated full-scale age-corrected IQ of 106.76 (*SD* = 13.57), and the neurotypical group had a score of 109.66 (*SD* = 8.43), which did not significantly differ, *t*(78) = 1.21, *p* = .25, *d* = 0.26. No differences were found between participants with ADHD who were not medicated and those with ADHD who were medicated on full-scale age-corrected IQ, *t*(36) = .58, *p* = .56, *d* = 0.19, performance on the Trail Making Task, *t*(36) = 1.62, *p* = .11, *d* = 0.53, or performance on the Stroop Color–Word Task, *t*(36) = 1.47, *p* = .15, *d* = 0.48. In addition, we did not find any significant differences in metacognitive ratings based on medication status. The educational attainment of the children’s parents was as follows: six had not completed high school, 14 had completed at least 1 year of college or university, 58 held a graduate or professional degree, and two did not report their educational attainment. The groups did not significantly differ in gender distribution or age. Data from this sample are also reported in Ledochowski et al. (2019) and Basile et al. (2021).

### Measures

#### Screening and Diagnostic Measures

##### Computerized Diagnostic Interview Schedule for Children—Parent Version (C-DISC)

The C-DISC is a computerized structured interview that is used to assess DSM-IV psychiatric disorders, symptoms, and level of impairment in children and adolescents aged 6 to 17 years of age (Fisher et al., 2006). Because C-DISC symptom scoring closely parallels DSM criteria and the primary change from DSM-IV to DSM-5 for ADHD is the age-of-onset requirement, children classified as meeting or not meeting criteria on the C-DISC would be expected to receive the same diagnostic status under DSM-5-TR. The Attention/Deficit-Hyperactivity Disorder (ADHD), Oppositional Defiant Disorder (ODD), and Conduct Disorder (CD) subscales were administered to parents by trained clinical psychology graduate students who were supervised by a registered psychologist. Parents answered questions about whether their child experienced specific symptoms over the past year, followed by questions about the noted symptoms. The ADHD scale was specifically used as a diagnostic screener for ADHD.

##### Child Behavior Checklist (CBCL)

The CBCL is a widely used and well-validated measure of behavioral and emotional outcomes in children and adolescents aged 6 to 18 years, completed by caregivers (Achenbach & Rescorla, 2001). This measure includes syndrome scales and updated DSM-5-oriented scales. Each item was scored on a three-point scale ranging from *0 = Absent, 1 = Occurs Sometimes, and 2 = Occurs Often.* For an item to be endorsed, it must have occurred within the last 6 months. The DSM ADHD scale was used to validate clinical group assignment.

#### Cognitive Tasks

##### Kaufman Brief Intelligence Test, Second Edition (KBIT-2)

The KBIT-2 is a brief assessment of intellectual ability, measuring both verbal and nonverbal abilities (Kaufman & Kaufman, 2004). Verbal intelligence was assessed using two subtests: Verbal Knowledge (language and general knowledge) and Riddles (verbal reasoning and comprehension). Nonverbal intelligence was assessed using a Matrices subtest, which measures abstract reasoning. The verbal and nonverbal intelligence scores were used to compute both age-corrected and non-age-corrected composite IQ scores. The age-corrected score was used to provide an IQ estimate for characterizing the sample. The non-age-corrected composite intelligence scores were obtained by summing the standardized z-scores of each composite raw score and were used in our analyses. Higher standardized scores indicate higher intellectual abilities.

##### Trail-Making Test (TMT)

The TMT is an executive function task that provides a measure of set-shifting, the ability to display flexibility when there are changing rules/schedules of reinforcement in the environment (Reitan, 1971; Strauss et al., 2006). The task involves two components: (1) Part A requires joining the digits 1-25 in numerical order with a pencil; (2) Part B requires participants to connect alternating letters and numbers in alpha-numerical order (i.e., 1 to A, A to 2, 2 to B and so forth). Part B contains 13 numbered and 12 lettered circles. The dependent measure for this study was the amount of time to complete Part B minus the amount of time to complete Part A (Strauss et al., 2006). Higher completion times indicate lower set-shifting abilities.

##### Stroop Color-Word Test

The Stroop test is an executive function task that measures interference control, the ability to filter irrelevant and relevant information (Friedman & Miyake, 2004; Golden, 1978). The task consisted of three conditions, each comprising 48 stimulus items arranged in a 6x8 matrix. In the word reading condition, participants had to read color words (red, blue, green, yellow) presented in black ink. In the color naming condition, participants named patches of color (red, blue, green, yellow). In the interference condition, participants were presented with words printed in various ink colors. The words were printed in an incongruent ink color (i.e., the word red was printed in yellow ink). Participants were asked to name the color of the ink and ignore the written word. In each condition, participants were asked to read the stimuli (colors or words) as quickly as possible without making any errors. The dependent variable used in this study was the total naming time for the interference condition, minus the total naming time for the color naming condition (Strauss et al., 2006). Higher interference times indicate difficulties in inhibition.

##### Unstructured Performance Task (UPT)

This task involves completing several simple questions with minimal structure and minimal direction provided by the examiner (Ledochowski et al., 2019). It was developed for children aged 8 to 12 years of age (Ledochowski et al., 2019). This task was presented on an 11x17-inch sheet of paper, which contained 42 simple questions on math, reading, general knowledge, and questions that involved direct copying (see Appendix). The items are placed on the paper in a random and unstructured manner. For the items to which children did not know the answer, they were instructed to circle that item. This was done to control task difficulty. The instructions provided were as follows: “I would like you to complete the following worksheet. If you do not know the answer for any of the problems, just circle it and go on to the next problem. I cannot read any of the questions to you. Just do your very best, and when you are done, please bring the worksheet to me.” Participants were allowed a maximum of 10 minutes to complete the task. The participants were not aware that the task would be discontinued after 10 minutes. Participants were scored on total number of correct, incorrect, circled, and blank items. Cronbach’s α for this task was .94, as reported for this dataset in Ledochowski et al. (2019). The total number of correct items was used as a dependent measure, with higher scores indicating better task performance.

#### Metacognitive Ratings

After participants completed each of the performance-based measures (KBIT-2, Trail Making Task, Stroop Task, and UPT), they were asked to rate the difficulty of each task on a 5-point scale, anchored by visual depictions of a figure carrying a heavy box (*4 = Heavy load*) or a lighter load (*0 = Light load*; see Appendix). Participants also rated how hard they tried on each task using a 5-point scale (*1 = I did not try at all*, *5 = I tried very hard*; see Appendix). Higher scores reflected greater perceived task difficulty and higher self-reported effort. These ratings served as the metacognitive dependent measures for each task.

### Procedure

Two trained examiners met with each child and their parent(s). They obtained informed consent and assent. One examiner administered measures to the child and the other to the parent. Testing and assessments took 90 to 120 minutes to complete. Each participant received compensation of $20. The institutional research ethics board approved this study.

### Statistical Analyses

All statistical analyses were conducted using IBM SPSS version 29.0. The level of significance for all analyses was set to *p* < .05. We conducted independent-samples *t*-tests for group comparisons on descriptive variables, mixed repeated-measures ANOVAs to examine group and task effects on metacognitive ratings, and Pearson correlations to evaluate associations between task performance and metacognitive ratings. Normality of the key variables was assessed using the Shapiro–Wilk test, along with visual inspection of histograms and Q–Q plots. Several variables showed significant negative skewness, indicating non-normal distributions with longer left tails. Performance measures exhibited significant skew, including Trail Making (skewness = −1.14, SE = 0.14), Stroop (skewness = −0.84, *SE* = 0.14), and UPT (skewness = −1.89, *SE* = 0.14). KBIT-2 scores did not show significant skew (skewness = −0.15, *SE* = 0.14). Metacognitive ratings also showed significant skewness, with try ratings ranging from −0.84 to −1.95 and difficulty ratings ranging from −0.70 to 0.45. Parallel non-parametric tests (Spearman correlations) produced the same pattern of results, supporting the robustness of the correlational findings. Likewise, the sphericity-corrected repeated-measures tests (Greenhouse–Geisser and Huynh–Feldt) produced the same significant effects and comparable effect sizes as the standard mixed repeated-measures ANOVA results, indicating that the findings were robust to violations of normality and sphericity.

One parent of a neurotypical participant did not complete the C-DISC; in this case, the CBCL was used to determine inclusion in the neurotypical group. Data for three neurotypical participants were missing on the C-DISC and CBCL; data were imputed based on group means. One participant with ADHD was missing both metacognitive ratings on the UPT; these values were also imputed using group means. Multiple comparisons for post-hoc pairwise tests in the mixed repeated-measures ANOVAs and for the correlation matrices were controlled using the Holm–Bonferroni adjustment. Descriptive group comparisons in Table 1 are based on raw scores to maintain information for sample characterization.

**Table 1.** Group Differences in Clinical Characteristics, Performance, and Metacognitive Ratings.

Variable	ADHD (*n* = 38)	Neurotypical (*n* = 42)	*t*(78)	Cohen’s *d* effect size
Intellectual Ability				
KBIT-2 *z*-score (not age corrected)	−.17 (1.94)	.28 (1.10)	1.29	0.29
KBIT-2 *Task difficulty rating*	1.87 (1.07)	1.66 (1.07)	−0.84	0.19
KBIT-2 *How hard tried rating*	3.71 (1.04)	4.40 (0.83)	3.32***	0.74
Executive Function Tasks				
Trail-Making Part B – Part A time (s)	115.58 (68.89)	82.09 (43.69)	−2.62*	0.59
Trail-Making *Task difficulty rating*	1.86 (1.25)	1.93 (1.07)	0.23	0.05
Trail-Making *How hard tried rating*	3.89 (1.22)	4.59 (0.80)	3.06**	0.68
Stroop Interference Time (s)	52.21 (23.98)	41.29 (15.60)	−2.44*	0.55
Stroop *Task difficulty rating*	2.52 (1.18)	2.57 (1.21)	0.17	0.04
Stroop *How hard tried rating*	4.26 (1.08)	4.59 (0.77)	1.59	0.36
Unstructured Performance Task (UPT)				
UPT (Correct raw score)	32.00 (8.84)	38.04 (3.33)	4.12***	0.92
UPT *Task difficulty rating*	1.73 (1.41)	1.02 (1.09)	−2.54*	0.57
UPT *How hard tried rating*	3.84 (1.28)	4.40 (0.94)	2.27*	0.51
CBCL ADHD total raw score	9.06 (2.46)	1.78 (1.91)	−14.87***	3.31
CBCL anxiety total raw score	5.78 (4.16)	2.11 (3.07)	−4.51***	1.01
CBCL depression total raw score	4.81 (4.38)	0.97 (1.80)	−5.03***	1.16

*Note.* Values are means (SDs). ADHD = Attention-Deficit/Hyperactivity Disorder; KBIT-2 = Kaufman Brief Intelligence Test, Second Edition; UPT = Unstructured Performance Task; CBCL = Child Behavior Checklist.

* *p* < .05. **p < .01. ***p < .001.

## Results

### Group Differences and Associations Among Performance and Metacognitive Ratings

Table 1 provides the means and standard deviations of performance and ratings for each group. All values in this table are raw, untransformed scores. Two separate mixed repeated-measures ANOVAs were conducted: one for ratings of how hard participants tried and one for ratings of difficulty across the four performance tasks. Group (ADHD vs. neurotypical) served as the between-subjects factor, and Task served as the within-subjects factor.

There was a significant main effect of Task on try ratings, *F*(3, 234) = 3.88, *p* = .01, η²_p_ = .05, indicating that ratings differed across the four performance tasks. Post-hoc pairwise comparisons with Holm–Bonferroni adjustment revealed that try ratings for the Stroop Task were significantly higher than those for the KBIT-2 (*p* = .008, 95% CI [0.07, 0.67]) and the UPT (*p* = .036, 95% CI [0.01, 0.60]). No other pairwise comparisons reached significance after correction (all *ps* > .05). A significant main effect of Group also emerged, *F*(1, 78) = 11.04, *p* < .001, η²_p_ = .12, such that the ADHD group reported lower overall try ratings across tasks compared to the neurotypical group. However, there was no significant interaction between Group and Task, *F*(3, 234) = 1.08, *p* = .36.

For the difficulty ratings, there was a significant main effect of Task, *F*(3, 234) = 19.57, *p* < .001, η²_p_ = .20, indicating that difficulty ratings differed across the performance tasks. Post-hoc pairwise comparisons with Holm–Bonferroni adjustment revealed that ratings for the Stroop Task were significantly higher than those for the KBIT-2 (*p* < .001, 95% CI [0.35, 1.21]), the Trail-Making Task (*p* < .001, 95% CI [0.30, 1.00]), and the UPT (*p* < .001, 95% CI [0.72, 1.62]). No other pairwise comparisons reached significance after correction (all *p*s > .05). The interaction effect between Task and Group was not significant, *F*(3, 234) = 2.63, *p* = .051, η²_p_ = .03, indicating that group differences in difficulty ratings did not vary across tasks. There was also no significant main effect of Group on overall difficulty ratings, *F*(1, 78) = 1.25, *p* = .27, η²_p_ = .02.

Pearson correlations among the performance tasks (KBIT-2, Trail-Making Task, Stroop Task, and UPT) and metacognitive ratings (try and difficulty) for the full sample are presented in Table 2. Corresponding within-group correlations for the ADHD and neurotypical groups are provided in the Appendix.

**Table 2.** Correlations Among Task Performance and Metacognitive Ratings in the Total Sample.

Variable	1	2	3	4	5	6	7	8	9	10	11
1. KBIT-2 z-score (not age corrected)	-										
2. KBIT-2 difficulty rating	.05	-									
3. KBIT-2 try rating	.11	.11	-								
4. Trail-Making Part B – Part A time (s)^a^	.39*	−.14	.06	-							
5. Trail-Making difficulty rating	−.15	.26	.03	−.21	-						
6. Trail Making try rating	.08	−.06	.46*	.03	.33	-					
7. Stroop Interference Score (s)^a^	.33	−.13	.14	.41*	−.03	.12	-				
8. Stroop difficulty rating	.09	.21	.18	.14	.51*	.15	−.07	-			
9. Stroop try rating	.21	−.11	.45*	.05	.10	.61*	.17	.36	-		
10. UPT total correct	.49*	−.10	.12	.66*	−.19	−.01	.49*	.09	.06	-	
11. UPT difficulty rating	−.41*	.18	−.17	−.12	.32	−.12	−.26	.25	−.03	−.37*	-
12. UPT try rating	−.09	−.06	.45*	−.04	−.05	.43*	.21	.02	.58*	−.08	.10

*Note.* KBIT-2 = Kaufman Brief Intelligence Test, Second Edition; UPT = Unstructured Performance Task.

a Scores were reflected such that higher scores indicate better performance.

* Remain significant at *p* < .05 after applying Holm–Bonferroni correction.

Within the full sample, task performances were positively correlated and generally significant. UPT correct raw scores were significantly associated with the KBIT-2 (*r* = .49, *p* < .001), Stroop Task (*r* = .49, *p* < .001), and Trail-Making Task (*r* = .66, *p* < .001). KBIT-2 scores were significantly associated with Trail-Making performance (*r* = .39, *p* < .001), although the correlation with the Stroop Task did not remain significant after Holm-Bonferroni correction (*r* = .33, *p* = .003). Trail-Making performance was significantly associated with Stroop performance (*r* = .41, *p* < .001).

Associations among the metacognitive try ratings were all positive and statistically significant after Holm-Bonferroni correction, with correlations ranging from *r* = .43 to *r* = .61 (all *p*s < .001). Specifically, correlations for try ratings were *r* = .46 between KBIT-2 and Trail-Making Task, *r* = .45 between KBIT-2 and Stroop Task, *r* = .45 between KBIT-2 and UPT, *r* = .61 between Trail-Making Task and Stroop Task, *r* = .43 between Trail-Making Task and UPT, and *r* = .58 between UPT and Stroop Task. In contrast, correlations among the difficulty ratings were weaker overall, ranging from *r* = .18 to *r* = .51. Specifically, correlations for difficulty ratings were *r* = .26 between KBIT-2 and Trail-Making Task, *r* = .21 between KBIT-2 and Stroop Task, *r* = .18 between KBIT-2 and UPT, *r* = .51 between Trail-Making Task and Stroop Task, *r* = .32 between Trail-Making Task and UPT, and *r* = .25 between Stroop Task and UPT. After Holm-Bonferroni correction, only the association between the Trail-Making Task and the Stroop Task remained significant (*r* = .51, *p* < .001). This pattern suggests that try ratings may reflect a more trait-like, person-level tendency, such that individuals who report trying hard on one task tend to report trying hard across tasks. Difficulty ratings, in contrast, appear to be more task-specific, which may explain why the strongest and only significant association after correction emerged between two tasks that both conceptually measure EF.

Correlations between task performance and their corresponding try ratings were all non-significant, ranging from *r* = -.08 to *r* = .17 (all *p*s > .05). Specifically, the correlations were *r* = .11 for KBIT-2, *r* = .03 for Trail-Making Task, *r* = .17 for Stroop Task, and *r* = −.08 for UPT. A similar pattern was observed for difficulty ratings: correlations between task performance and their corresponding difficulty ratings were *r* = .05 for the KBIT-2, *r* = −.21 for the Trail-Making Task, *r* = −.07 for the Stroop Task, and *r* = −.37 for the UPT, with only the association between UPT difficulty ratings and UPT performance being significant after Holm-Bonferroni correction (*r* = −.37, *p* < .001). Additionally, UPT difficulty ratings were also associated with performance on the KBIT-2 (*r* = −.41, *p* < .001). Finally, within-task associations between try and difficulty ratings were all non-significant after Holm–Bonferroni correction, with correlations ranging from *r* = .10 to *r* = .36. Specifically, correlations between try and difficulty ratings were *r* = .11 for the KBIT-2, *r* = .33 for the Trail-Making Task, *r* = .36 for the Stroop Task, and *r* = .10 for the UPT. Taken together, these findings indicate that metacognitive try and difficulty ratings are largely independent of objective task performance and represent conceptually distinct aspects of subjective effort.

## Discussion

The purpose of this study was to examine whether metacognitive ratings of task difficulty and effort (i.e., how hard one tried) differed across several cognitive tasks in children with ADHD and neurotypical controls. In particular, these ratings provide insight into whether subjective metacognitive judgments further inform our understanding of performance differences on cognitive tasks, such as measures of intelligence and executive functions, in children with ADHD (Frazier et al., 2004; Willcutt et al., 2005). Children with ADHD reported lower overall ratings of how hard they tried across the tasks compared to the neurotypical group, but no overall group differences in ratings of task difficulty. In the total sample, ratings of how hard participants tried were more consistently positively intercorrelated across tasks than ratings of task difficulty. Metacognitive ratings of how hard one tried and task difficulty were generally not associated with accuracy on tasks in the total sample. Finally, effort and task difficulty ratings for each respective task were not significantly correlated, consistent with the view that these judgments reflect separable metacognitive processes.

### ADHD Versus Neurotypical Group Differences on Metacognitive Ratings

Children with ADHD reported lower ratings of “how hard I tried” across the four performance tasks relative to the neurotypical group. However, there was no significant effect of task type on these group differences in ratings, suggesting that lower perceived effort reflects a more general, cross-task tendency rather than being tied to specific task parameters. A significant effect of task did emerge, with children in both groups reporting higher try ratings on the Stroop Task compared to the KBIT-2 and the Unstructured Performance Task, but not compared with the Trail-Making Task, another EF measure. There were no overall group differences in ratings of task difficulty across the four performance tasks. Similar to findings for try ratings, children from both groups reported higher difficulty ratings on the Stroop Task compared to the other three tasks administered. Overall, these findings suggest that ratings of how hard one tried may more consistently differentiate participants with ADHD from neurotypical participants, regardless of the type of task, than ratings of task difficulty.

These group differences and the pattern of findings between ratings of task difficulty and how hard participants tried suggest that these metacognitive ratings are conceptually distinct, consistent with prior studies (Hoch et al., 2023; Schuessler et al., 2024). Ratings of task difficulty reflect the mental or cognitive demands of the task, whereas ratings of how hard one tried reflect volitional or applied effort, including both data-driven and goal-driven aspects of effort (Ackerman, 2014; Hoch et al., 2023; Koriat et al., 2006; Schmeck et al., 2015). Task difficulty ratings emphasize the characteristics of the task, particularly its level of difficulty relative to other tasks. In contrast, ratings of “how hard I tried” likely index the motivation and energy intentionally invested by an individual. Koriat (2018; Koriat et al., 2006, 2014; Koriat & Nussinson, 2009) distinguish between data-driven and goal-driven effort, which characterize different types of processing that may underlie these subjective ratings. Data-driven effort is described as bottom-up processing that reflects the effort required by the task itself, whereas goal-driven effort is described as top-down processing that reflects the deliberate effort allocated by the individual (Koriat et al., 2014). Our pattern of findings based on group differences is consistent with the separability of these metacognitive ratings and their associated processing mechanisms.

In addition, the relatively consistent differences in effort ratings (but not task difficulty) between neurotypical and ADHD participants point to differences in goal-driven rather than data-driven effort in individuals with ADHD. That is, individuals with ADHD may not differ from neurotypicals in how they perceive task requirements or demands, but they may differ in how much effort they exert to meet those demands. The lack of group differences in task difficulty ratings may partly reflect the commonly observed Positive Illusory Bias in ADHD (Owens et al., 2007), whereby individuals under-recognize their difficulties and rate their own functioning more favorably than would be expected based on objective performance. In this context, children with ADHD may downplay how challenging tasks feel, even when their performance is generally lower. While this pattern of findings helps further characterize difficulties associated with ADHD, it also raises questions that may be addressed by metacognitive models that offer testable hypotheses about the mechanisms linking monitoring and control. In particular, we might infer from the present study that individuals with ADHD do not differ in how they perceive task demands (no group differences in task difficulty ratings); rather, they differ in how they decide to approach the task or allocate resources to completing it (group differences in how hard they tried).

Metacognitive models can assist with operationalizing and separating monitoring and control of cognitive processing (Dunlosky & Metcalfe, 2009; Koriat & Goldsmith, 1996; Nelson, 1990). For example, changes in perceived task difficulty (monitoring) can signal individuals to adjust their behavior (control), such as altering the time spent on a problem, as posited in models like the Diminishing Criterion Model (DCM; Ackerman, 2014). Given that the DSM-5-TR diagnostic criteria for ADHD include a symptom related to effort engagement [“avoid, dislike, or are reluctant to engage in tasks that require sustained mental effort (e.g., schoolwork or homework; for older adolescents and adults, preparing reports, completing forms, reviewing lengthy papers)” (APA, 2022, p. 68)], metacognitive models can advance our understanding by further operationalizing the conditions under which these difficulties arise, such as identifying whether they reflect problems with monitoring or with control of metacognitive resources. Previous models of ADHD have suggested that individuals have difficulty allocating sufficient cognitive and emotional resources to tasks (e.g., Sergeant, 2005); however, recent developments in the measurement of mental effort can also advance and refine this literature (Wagner et al., 2024).

The group differences observed for how hard participants reported trying diverge from findings reported in prior studies, including Brown et al. (2020) and Mies et al. (2019). Notably, Mies et al. (2019) was a pilot study with a relatively small sample (15 boys with ADHD and 16 controls). In addition, their applied effort rating (“how much they did their best”) differed from the phrasing used in the current study (“how hard did you try on this task”). These wording differences may explain why the try ratings differed across studies and underscore the importance of examining how question phrasing may shape self-reported effort, such as the possibility of socially desirable responding. Despite the relatively small sample in the Mies et al. (2019) study, the absence of group differences in task difficulty ratings aligns with this study, perhaps partly attributable to the use of similar question phrasing with respect to the task difficulty rating. Mies et al. (2019) similarly reported no differences between ADHD and neurotypical groups in perceived cognitive demand.

With respect to whether task characteristics influenced metacognitive differences between groups, our findings indicate that task type did not moderate group differences in either difficulty or try ratings, given the relatively consistent findings across metacognitive rating types. This suggests that group differences in perceived effort and task difficulty were consistent across tasks varying in cognitive domain (EF vs. IQ) and structure (from the highly structured KBIT-2 to the unstructured UPT). However, across the total sample, the Stroop Task stood out as the task on which participants reported the highest ratings of both difficulty and how hard they tried, suggesting that it was experienced as more demanding than the other tasks. The Stroop Task relies heavily on inhibitory control, a cognitive process shown to be effortful and mentally fatiguing (Hagger et al., 2010).

### Associations Between Ratings of Task Difficulty and How Hard One Tried

Metacognitive ratings of how hard participants reported trying were positively, significantly, and consistently related across the four tasks in the total sample, indicating that children who reported trying hard on one task tended to report trying hard on the others. In contrast, associations among perceived task difficulty were generally small and often non-significant in the total sample, varying more across tasks. Metacognitive ratings were also generally not significantly correlated with accuracy on the cognitive tasks, except for the UPT difficulty ratings, which were negatively associated with UPT performance and KBIT-2 performance (i.e., higher perceived difficulty was related to poorer performance). Finally, ratings of how hard one tried and ratings of task difficulty within each task in the total sample were not significantly correlated, suggesting that these metacognitive judgments are separable.

Ratings of ‘how hard one tried’ were strongly and consistently associated across all performance tasks, consistent with Mulert et al. (2007), who found ratings of volitional effort (i.e., how hard I tried) to be constant across variations in task difficulty. In contrast, task difficulty ratings showed weaker, mostly non-significant correlations, with the only significant association between task difficulty ratings emerging for tasks that assessed similar EF constructs (Stroop Task and Trail-Making Task). This pattern is consistent with the view that try ratings capture a more trait-like, person-level tendency to invest effort across tasks, whereas difficulty ratings are more tightly linked to the specific demands of a given task (e.g., Hoch et al., 2023; van Gog & Paas, 2008). In addition, the differences in the pattern of correlations for the task difficulty and effort ratings in the total sample are also consistent with the conceptual distinction between these metacognitive ratings (Hoch et al., 2023).

Finally, there was a lack of significant associations between metacognitive ratings and task performance, with the exception of a significant negative association between ratings of task difficulty on the UPT and both UPT total correct raw score and KBIT-2 performance. Overall, these patterns contrast with findings by Hoch et al. (2023), who reported strong item-level associations between perceived difficulty and accuracy across different performance tasks. However, these differences are likely attributable to a key methodological distinction: their difficulty ratings were collected at the item level, whereas the present study relied on global post-task ratings.

Similar to trait-based scales such as the Barkley Deficits in Executive Functioning Scale for Children and Adolescents (BDEFS-CA; (Barkley, 2012) and other rating scales commonly used in the assessment of ADHD (e.g., Behavior Rating Inventory of Executive Function [BRIEF]; Gioia et al., 2000), the metacognitive ratings examined in the current study are also subjective, like the BDEFS-CA. However, an important difference is that the ratings in the present study were anchored to specific cognitive tasks that had just been completed. In addition, the metacognitive ratings were completed by the children themselves who performed the tasks, rather than by a parent informant, as is typical for the BDEFS-CA and other EF rating scales. These findings suggest the potential for metacognitive ratings to contribute to ADHD assessment in a way that is analogous to EF rating scales, while uniquely indexing monitoring processes in relation to a specific task. Although further research is needed, metacognitive ratings may provide more nuanced information about the sources of difficulty on these tasks, such as distinguishing problems in monitoring from problems in control. This, in turn, may inform intervention strategies and contribute to the emerging literature on interventions targeting metacognitive perceptions and evaluations in children with ADHD (Lenartowicz et al., 2024).

Future studies should include measures with more items and metacognitive ratings for each item, or obtain ratings multiple times during a task, as multiple ratings may importantly differ from single-item post-task effort ratings (van Gog et al., 2012) and provide the opportunity to assess other metacognitive indices, such as calibration and resolution. Additionally, future studies should also incorporate measures of participants’ perceived task performance to determine whether suspecting poor performance might trigger a self-protective mechanism, manifesting in lower self-reported effort and/or higher perceived task difficulty (Diener & Milich, 1997; Owens et al., 2007). In addition, our sample is comprised primarily of males, which may limit the generalizability of our findings to females. Previous research has provided some evidence of differences in metacognitive ratings between boys and girls with ADHD, also relative to their neurotypical peers (Hoza et al., 2004; Owens & Hoza, 2003). One of the core inattention symptoms in ADHD is avoiding or disliking tasks that require sustained mental effort (DSM-5-TR; APA, 2022), which is conceptually close to the effort ratings examined in the present study. There is evidence that girls with ADHD show higher levels of internalizing problems and lower self-efficacy and coping than boys with ADHD (Rucklidge, 2001, 2010), which may shape how they interpret and report effortful engagement on cognitive tasks. As a result, girls with ADHD might show different patterns of self-reported effort and task difficulty, or different links between these ratings and objective performance, than the largely male ADHD sample included here. Future work with more gender-balanced samples is needed to test gender as a moderator of effort and difficulty ratings in ADHD.

Children in the ADHD group consistently reported lower effort ratings across the cognitive tasks compared to neurotypical children, despite showing no overall group differences in perceived task difficulty. Effort ratings were strongly related across tasks, suggesting that they may reflect a more trait-based tendency, whereas task difficulty ratings showed weaker cross-task associations, suggesting that these ratings were more influenced by task-specific factors. These findings highlight that brief, task-anchored metacognitive ratings of effort (specifically “how hard I tried”) may further operationalize the types of difficulties experienced by children with ADHD, and that using metacognitive models to generate further testable hypotheses may provide insight into the sources of these difficulties, such as monitoring and control processing. Given that commonly used neuropsychological and EF measures, such as those administered in this study, have been found to show no incremental validity for ADHD diagnosis beyond symptom reports and related clinical information (Sawaya et al., 2024), task-anchored effort ratings may provide diagnostically relevant information with respect to the attentional and self-regulatory difficulties in ADHD within structured testing contexts.

## Figures and Tables

**Figure A1.**
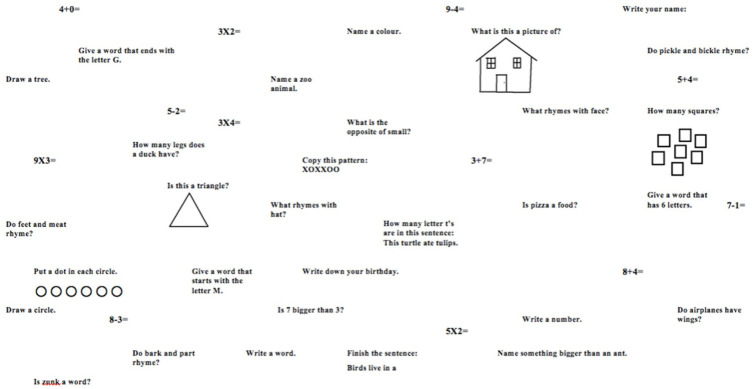
Unstructured performance task sample.

**Figure A2.**
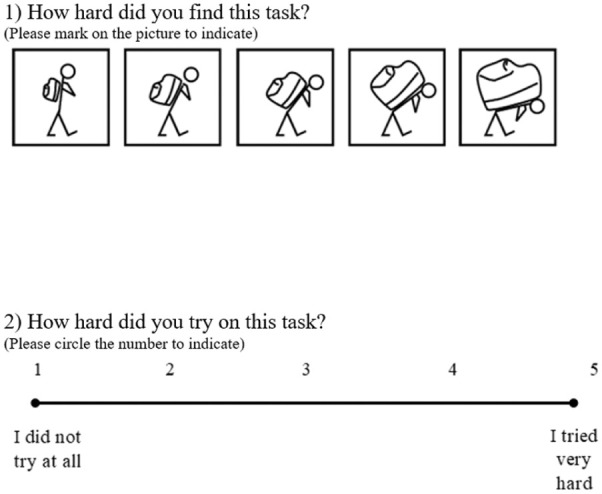
Metacognitive ratings.

## Appendix

**Table A1.** Correlations Between Task Performance and Metacognitive Ratings in the Neurotypical Group (*N* = 42).

Variable	1	2	3	4	5	6	7	8	9	10	11
1. KBIT-2 z-score (not age corrected)	-										
2. KBIT-2 difficulty rating	.24	-									
3. KBIT-2 try rating	.07	.21	-								
4. Trail-Making Part B – Part A time (s)^a^	.01	.10	−.09	-							
5. Trail-Making difficulty rating	.10	.36	.25	−.20	-						
6. Trail Making try rating	.10	.27	.48	−.17	.42	-					
7. Stroop Interference Score (s)^a^	.20	.08	.04	.14	.10	−.04	-				
8. Stroop difficulty rating	.09	.34	.30	.01	.67*	.40	−.19	-			
9. Stroop try rating	−.01	.25	.65*	−.27	.44	.76*	−.08	.49	-		
10. UPT total correct	.41*	.30	−.11	.44	.00	−.15	.19	.20	−.18	-	
11. UPT difficulty rating	−.34	.28	−.01	−.03	.38	.23	−.29	.34	.22	−.21	-
12. UPT try rating	−.15	.23	.57*	−.34	.32	.61*	−.01	.16	.64*	−.28	.18

*Note.* KBIT-2 = Kaufman Brief Intelligence Test, Second Edition; UPT = Unstructured Performance Task.

a Scores were reflected such that higher scores indicate better performance.

* Remain significant at *p* < .05 after applying Holm–Bonferroni correction.

**Table A2.** Correlations Between Task Performance and Metacognitive Ratings in the ADHD Group (*N* = 38).

Variable	1	2	3	4	5	6	7	8	9	10	11
1. KBIT-2 z-score (not age corrected)	-										
2. KBIT-2 difficulty rating	−.04	-									
3. KBIT-2 try rating	.06	.11	-								
4. Trail-Making Part B – Part A time (s)^a^	.52	−.27	−.02	-							
5. Trail-Making difficulty rating	−.29	.17	−.15	−.24	-						
6. Trail making try rating	.01	−.24	.34	−.03	.29	-					
7. Stroop interference Score (s)^a^	.35	−.25	.06	.46	−.13	.08	-				
8. Stroop difficulty rating	.09	.08	.08	.24	.36	−.02	.01	-			
9. Stroop try rating	.29	−.37	.29	.13	−.13	.51	.23	.27	-		
10. UPT total correct	.50	−.19	−.02	.68*	−.32	−.18	.52	.05	.03	-	
11. UPT difficulty rating	−.42	.06	−.12	−.05	.30	−.18	−.16	.20	−.10	−.33	-
12. UPT try rating	−.12	−.27	.29	−.01	−.32	.26	.25	−.11	.51	.05	.19

*Note.* KBIT-2 = Kaufman Brief Intelligence Test, Second Edition; UPT = Unstructured Performance Task.

a Scores were reflected such that higher scores indicate better performance.

* Remain significant at *p* < .05 after applying Holm–Bonferroni correction.
